# Complete and Closed Genome Sequences of 10 *Salmonella enterica* subsp. *enterica* Serovar Anatum Isolates from Human and Bovine Sources

**DOI:** 10.1128/genomeA.00447-16

**Published:** 2016-06-02

**Authors:** Scott V. Nguyen, Dayna M. Harhay, James L. Bono, Timothy P. L. Smith, Patricia I. Fields, Blake A. Dinsmore, Monica Santovenia, Christy M. Kelley, Rong Wang, Joseph M. Bosilevac, Gregory P. Harhay

**Affiliations:** a USDA ARS U.S. Meat Animal Research Center, Clay Center, Nebraska, USA; b Enteric Diseases Laboratory Branch, Division of Foodborne, Waterborne and Environmental Diseases, Centers for Disease Control and Prevention, Atlanta, Georgia, USA

## Abstract

*Salmonella enterica* is an important pathogen transmitted by numerous vectors. Genomic comparisons of *Salmonella* strains from disparate hosts have the potential to further our understanding of mechanisms underlying host specificities and virulence. Here, we present the closed genome and plasmid sequences of 10 *Salmonella enterica* subsp. *enterica* serovar Anatum isolates from bovine and human sources.

## GENOME ANNOUNCEMENT

Infections by *Salmonella enterica* (nontyphoidal) are one of the most common causes of foodborne illnesses, with over a million estimated cases in the United States and an economic impact of $3.66 billion in 2013 (1). The far-reaching impact of this pathogen can be attributed to the many niches and vectors it is able to occupy. Yet in spite of this source diversity, the majority of fully sequenced *S. enterica* isolates to date are of strains isolated from humans. Expanding the genomic purview of *Salmonella* from human food sources will facilitate epidemiological tracking as well as comparative genomic methods of source attribution. *Salmonella enterica* subsp. *enterica* serovar Anatum is frequently found to be associated with animals in production agriculture (2, 3), including cattle, and yet is not commonly attributed to human salmonellosis (4). Additional genomic and plasmid sequence data for *S*. Anatum will improve phylogenetic comparisons in different niches. Here, we announce the complete closed genome and plasmid sequences of 10 *S*. Anatum isolates from cattle (ground beef, hide, and pre-evisceration carcasses) and human salmonellosis.

DNA was isolated from overnight cultures grown at 37°C in Trypticase soy broth (Becton, Dickinson, Franklin Lakes, NJ) and was purified using Genomic-tip 100/G columns and a DNA isolation kit, as per the included protocol (Qiagen, Valencia, CA). Sequencing libraries for single-molecule real-time sequencing (SMRT) on a PacBio RS II instrument (Pacific Biosciences, Menlo Park, CA) were prepared as per the recommendation for P4-C2/P5-C3 chemistry. SMRT produced average subreads of >7 kb and mean coverage of 157×. Complete single-contig chromosomes were assembled using the Celera assembler version 7.0 (5) and then polished by Quiver (6). Use of a self/self-dot plot of consensus sequences in Geneious 8.1.6 (Biomatters Ltd., New Zealand) (7) revealed duplicated ends that were then trimmed at the 3′ end to generate a circularized sequence. Ori-Finder (8) was used to determine the origin of replication and to reset base position 1 of the chromosome.

The average size of the *S*. Anatum genomes in this report was 4.77 Mb, with a range of 4.64 to 4.95 Mb (Table 1). Five plasmids associated with *S*. Anatum were assembled from four strains reported here and one previously reported strain, ranging in size from 9,323 bp to >160 kb (Table 1). Isoforms of plasmid pSAN1-2010K-2577 were detected, due to the presence of a shufflon (9). The sequence of the predominant isoform (103.8 kb) is reported here.

**TABLE 1:** Chromosome and plasmid sequence accession numbers and additional information for 10 *Salmonella enterica* subsp. *enterica* serovar Anatum strains

Strain or plasmid	NCBI accession no.	Size (bp)	Coverage (×)	Antibiotic resistance phenotype^a^	Source of isolation
pSAN1-1735^b^	CP014707	101,118	273.8		Bovine PRE^c^
USMARC-1676	CP014620	4,676,958	125.7	PS	Ground beef
USMARC-1677	CP014663	4,836,394	113.0	PS	Ground beef
USMARC-1727	CP014621	4,894,060	125.3	PS	Bovine PRE
pSAN1-1727	CP014622	97,041	225.1		
USMARC-1728	CP014664	4,783,492	136.2	PS	Ground beef
USMARC-1736	CP014657	4,704,371	192.4	Am Ap F Ax C K S Su Te	Bovine hide
pSAN1-1736	CP014658	160,227	225.0		
CDC 06-0532	CP007211	4,667,736	111.5	PS	Human stool
CDC 06-0624	CP014659	4,946,108	262.0	Te	Human stool
pSAN1-06-0624	CP014660	9,323	369.7		
CDC 06-0855	CP014665	4,751,034	134.3	PS	Human stool
CDC 08-1092	CP014666	4,636,307	184.9	PS	Human stool
CDC 2010k-2577	CP014661	4,700,848	146.3	Su Sxt	Human stool
pSAN1-2010K-2577	CP014662	103,851	288.2		

a PS, pansusceptible; Am, amoxicillin-clavulanic acid; Ap, ampicillin; F, cefoxitin; Ax, ceftriaxone; C, chloramphenicol; K, kanamycin; S, streptomycin; Su, sulfisoxazole; Sxt, sulfamethoxazole-trimethoprim; Te, tetracycline.

b Host strain USMARC-1735 was previously submitted to NCBI GenBank (10).

c PRE, pre-evisceration carcass.

### Nucleotide sequence accession numbers.

Genome and plasmid sequence data of the 10 *S*. Anatum isolates were annotated using the NCBI Prokaryotic Genome Annotation Pipeline and deposited into NCBI GenBank; the accession numbers are listed in Table 1.
